# Contrasting Effects of Long-Term Grazing and Clipping on Plant Morphological Plasticity: Evidence from a Rhizomatous Grass

**DOI:** 10.1371/journal.pone.0141055

**Published:** 2015-10-27

**Authors:** Xiliang Li, Zinian Wu, Zhiying Liu, Xiangyang Hou, Warwick Badgery, Huiqin Guo, Qingshan Zhao, Ningning Hu, Junjie Duan, Weibo Ren

**Affiliations:** 1 National Forage Improvement Center, Key Laboratory of Grassland Resources and Utilization of Ministry of Agriculture, Institute of Grassland Research, Chinese Academy of Agricultural Sciences, Hohhot, Inner Mongolia, 010010, P.R. China; 2 New South Wales Department of Primary Industries, Orange Agricultural Institute, Orange, New South Wales, 2800, Australia; 3 College of Life sciences, Inner Mongolia Agricultural University, Hohhot, Inner Mongolia, 010010, P.R. China; Institute of Genetics and Developmental Biology, Chinese Academy of Sciences, CHINA

## Abstract

Understanding the mechanism of plant morphological plasticity in response to grazing and clipping of semiarid grassland can provide insight into the process of disturbance-induced decline in grassland productivity. In recent studies there has been controversy regarding two hypotheses: 1) grazing avoidance; and 2) growth limiting mechanisms of morphological plasticity in response to defoliation. However, the experimental evidence presented for the memory response to grazing and clipping of plants has been poorly reported. This paper reports on two experiments that tested these hypotheses in field and in a controlled environment, respectively. We examined the effects of long-term clipping and grazing on the functional traits and their plasticity for *Leymus chinensis* (Trin.) Tzvelev (the dominate species) in the typical-steppe grassland of Inner Mongolia, China. There were four main findings from these experiments. (i) The majority of phenotypic traits of *L*. *chinensis* tended to significantly miniaturize in response to long-term field clipping and grazing. (ii) The significant response of morphological plasticity with and without grazing was maintained in a hydroponic experiment designed to remove environmental variability, but there was no significant difference in *L*. *chinensis* individual size traits for the clipping comparison. (iii) Plasticity indexes of *L*. *chinensis* traits in a controlled environment were significantly lower than under field conditions indicating that plants had partial and slight memory effect to long-term grazing. (iv) The allometry of various phenotypic traits, indicated significant trade-offs between leaf and stem allocation with variations in plant size induced by defoliation, which were maintained only under grazing in the hydroponic controlled environment experiment. Taken together, our findings suggest that the morphological plasticity of *L*. *chinensis* induced by artificial clipping was different with that by livestock grazing. The miniaturization of plant size in long-term grazed grassland may reflect retained characteristics of dwarf memory for adaptation to long-term grazing by large herbivores.

## Introduction

Grasslands occupy more than a third of the world’s land area, excluding Antarctica and Greenland, and support the livelihoods of approximately 1 billion people [1]. In the past 50 years, many of these grasslands, particularly in the Inner Mongolia Autonomous Region of northern China, have become degraded, affecting not only productivity but also the vital environmental services of ecosystems such as hydrology, biodiversity, and carbon cycles [2]. Human activity, has induced high grazing pressure by domestic livestock, which is recognized as the a primary cause of grassland degradation [3]. Plant functional traits are the features that represent ecological strategies and determine how plants respond to environmental factors, affect other trophic levels and influence ecosystem properties [4]. Until recently we have had relatively little knowledge about the response of grassland plant traits to over utilization [5] compared to responses at the landscape, ecosystem, community and population levels [3, 6] and in recent years there has been a focus on the response of functional traits to defoliation.

Changes in plant functional traits, can effectively signify shifts in ecosystem functions and processes, and can be more sensitive to disturbance from defoliation, than other ecological processes, such as community succession and biodiversity loss [7, 8]. Generally speaking, the leaf is the most important functional organ of the plant, and there are leaf traits (e.g. leaf hardness) that protect the plant from defoliation, thereby contributing to its stability in aboveground biomass of individual plants [9, 10]. Compared with un-grazed native grassland, the specific leaf area (SLA) would increase [11] and thereby improving the photosynthetic ability [12] in response to long-term grazing. Moreover, the sensitivity of plants is greater for the aboveground portion than the belowground portion [9, 13]. Root traits lag in response to defoliation compared with changes in the soil microenvironment, such as microorganisms, physiochemical properties and moisture [14–16]. Different plant species respond in different ways to grazing [17]. Taller plants had leaves with lower SLA; short species of intermediate toughness were selected more often by sheep; and short species with high SLA increased with grazing [18]. In addition, the ecological strategies (such as tradeoffs, allometry) of functional traits were adopted in optimizing to finish its growth cycle in an adverse habitat caused by defoliation [10, 19, 20]. Specifically, our previous study indicated that the functional traits of *Leymus chinensis* (Trin.) Tzvelev, the dominate species in eastern Eurasian temperate grassland, can adapt to long-term grazing by leaf and stem allometry [21].

Only a small number of ecological studies have investigated the effects of clipping on plant traits in typical steppe in eastern Eurasian temperate grassland [22, 23]. Differences in plant functional traits responding to clipping are extremely rare [24]. In several recent studies that have compared the response of functional traits to defoliation by clipping [25], clipping reduced the root to shoot ratio and increased specific root length [26, 27], but had no effect on specific root respiration [26]. Although there were no consistent conclusions in plant traits and tradeoffs under clipping according to Herrero-Jáuregui et al. [28]. Chen et al (2014) did find that clipping reduced the expression levels of ribosomal protein genes, cell division or cell expansion-related genes, and lignin biosynthesis genes which may have negatively affected the growth of *L*. *chinensis* [29]. However, in general, there is a dearth of information on the effects of long-term clipping on the growing ability of grassland plants, such as the dominant *L*. *chinensis*, in the eastern Eurasian temperate grassland.

Understanding the mechanism of plant morphological plasticity and tradeoffs between different traits in response to defoliation has long been a challenge for ecologists. Although ecological theories have provided different explanations for this scientific problem [30–32], there is on-going controversy concerning two main hypotheses about the mechanism of plant morphological plasticity in response to defoliation. The first is grazing avoidance hypothesis where it is thought that smaller plants can avoid the selective intake of animals by genetic modification (especially at the epigenetic level) [31–33]. The second is the growth limitation hypothesis, which rejects the genetic modification path and proposes that the plant morphological plasticity and tradeoffs between different traits were primarily caused by the altering of soil microenvironment such as fertility, hydrology and soil structure [10, 30, 34]. Many reports have showed that grazing stress can alter the physiochemical substance (e.g. soluble sugar, antioxidant substance and leaf photosynthetic capacity) [35, 36] and gene expression of grassland plants [37, 38]. These physiological, biochemical and molecular changes can help the plants to develop the adaptive phenotype change to response to grazing stress. The controversy between the two hypotheses stems from a lack of specific experimental evidence.

We tested the hypotheses in two experiments under field and laboratory conditions by examining the effects of clipping and grazing on the functional traits of *L*. *chinensis*, a dominate grass species in northern China [39] that reproduces by rhizomes [40]. This work was designed to address the following three questions: (i) How do *L*. *chinensis* traits respond when defoliated by grazing and clipping? (ii) Is there a similar response of functional traits to clipping and grazing? and (iii) To what degree does resource limitation cause *L*. *chinensis* morphological plasticity induced by defoliation?

## Materials and Methods

### Ethics statement

The field survey site was owned and/or managed by the Inner Mongolia Grassland Ecosystem Research Station (IMGERS) of Chinese Academy of Sciences and local pastoral farmers who gave permission to undertake the field research. Regarding to the field study, no specific permits were required for the *L*. *chinensis* species in the described locations, and the field studies did not involve endangered or protected species.

### Field Site Description

The study site is located at IMGERS (43°38’ N, 116°42’ E), in the Xilin River catchment, China at an altitude of ~1,200 m a.s.l. The semiarid continental climate is characterized by mean annual (1980–2012) precipitation of 258.73 mm and a mean annual temperature of 2.98°C. Typically, maximum precipitation coincides with highest temperatures in June, July, and August. For perennial grass species the 150 day growing season lasts from April/May to September/October. *L*. *chinensis* is the dominant perennial grass species of the typical steppe grasslands. The major soil types of this region are calcic chestnuts and calcic chernozems[41].

### Experimental Design and Sampling

The experiment consisted of two comparisons: unrestricted grazing with an enclosure (ungrazed); and biannual clipping with enclosure (unclipped). The grazing exclusion plot has been fenced since 1983 for long-term ecological observation and research. The grazing plot (~200 ha in area) was located adjacent to the grazing exclusion plot and has been grazed by ~600 sheep and goats all year round for more than 30 years at the stocking rate of ~3 sheep unit per hectare. This is significantly higher than local stocking rate of 1.5 sheep unit per hectare needed to maintain grass-livestock balance, which was recommended by the local government. The long-term clipping and clipping exclusion (unclipped) plots were established in the same location in 1997. There was no disturbance in the unclipped plot. In the clipped plot, the aboveground portion of all the plant species was clipped to about six centimeters above the ground over the entire plot in early June and late August every year. At the time of each harvest, using a small hand-push lawn mower, the grass was cut to a stubble height of 6 cm over the entire plot.

Like most chronosequence studies, pseudo-replication and space-for-time substitution limitations [42–44] formed the basis of the design with five 20m × 20m replicated plots established along a transect as pairs within the long-term grazed and ungrazed treatments. The plots were randomly allocated within 30m of each other along the transect. Three 1 m × 1 m subplots were established in each plot for field investigation and sampling. On clipping and grazing plots temporary movable ex-closure cages were set up at each sampling point prior to clipping and grazing before the growing season in early April, 2013. The field sampling was carried out during 15–20 August 2013, corresponding to annual peak-standing biomass. Three *L*. *chinensis* individuals (tillers) were selected randomly in each 1m×1m quadrats. The tillers were clipped at ground level and taken to a laboratory where their morphological traits were measured (Table 1).

**Table 1:** All *Leymus chinensis* (Trin.) Tzvelev functional traits examined.

Functional traits classification	Full name	Shortened form	Unit
leaf traits	Leaf number	*LN*	\
	Leaf length	*LL*	cm
	Leaf width	*LW*	mm
	Leaf length / width ratio	*LLW*	cm/mm
	Total leaf area	*TLA*	cm^2^
	Averaged leaf area	*LA*	cm^2^
stem traits	Stem length	*SL*	cm
	Stem diameter	*SD*	mm
	Stem length / diameter ratio	*SLD*	cm/mm
whole-plant traits	Plant height	*PH*	cm

According to the standard methods [4], morphological traits of leaf length, leaf number, stem length, stem width, plant height, were measured using an electronic digital caliper (Table 1). The leaves digitally scanned (indicate device) and their leaf areas measured using Adobe Photoshop CS5.

### Laboratory Experiment

Since *L*. *chinensis* is a rhizomatous perennial grass that reproduces via clonal propagation by rhizome [40], we used asexual reproduction to test the effects of grazing and clipping on *L*. *chinensis* growth. After the end of growing season (October, 2013) *L*. *chinensis* rhizomes, which were at a similar stage of development, were sampled in both grazed and clipped treatments. The rhizomes were cut to 2 cm lengths and cultivated in a hydroponic environment to remove the influence of nutrition, water and light. Each rhizome with a bud was transferred to a hydroponic container with 1 × Hoagland nutrient solutions [45, 46] in a growth chamber. Six hydroponic containers of *L*. *chinensis* rhizomes were planted in each group. The hydroponic containers (10cm diameter × 50cm high) were randomly arranged in the growth chamber under the following conditions: 16 h photoperiod, 25°C daytime temperature, 15°C nighttime temperature and a relative humidity of 70–80%. The controlled environment was used ensure uniformity and to minimize uncontrolled sources of variation [47]. Artificial lighting was provided by a mixture of high-pressure sodium and metal halide 400 W lamps with a photosynthetic photon flux density of 550 umol photons m^−2^ s^−1^. In total, 24 hydroponic containers were randomly arranged in an incubator. After 95 days growing, samples of the mature plants were destructively sampled in all of the 24 hydroponic containers. Three *L*. *chinensis* were selected randomly and the functional traits (Table 1) were measured using the same method as the field experiment.

### Statistical Analysis

Statistical analyses were made on the average functional traits over the three individuals for each quadrat in the field experiment. A principal component analysis (PCA) was performed to determine relations among the 10 functional traits and the effect of grazing and clipping on these traits [48]. For this analysis we centred and normalised all variables with their standard deviations because they had different units. The importance of a trait in a given component is indicated by its relative loading on a component. The significance of these loadings was tested using Pearson's correlation test for all traits of *L*. *chinensis* individuals [49].

Significant differences in plant traits between the unclipped and clipped, and ungrazed and grazed plots were evaluated by one-way analysis of variance (ANOVA) procedures. Correlations among functional traits were analyzed by the Pearson method. The degrees of responses of *L*. *chinensis* functional traits between grazed and ungrazed grassland were analyzed by plasticity index (*PI*) [50].

$$PI=\frac{FU-FD}{FD}$$ (1)

where *FU* is functional traits in long-term unclipped or ungrazed habitats. *FD* is functional traits in long-term clipped or grazed habitats.

The partial memory effects of grazing and clipping on the morphological plasticity of *L*. *chinensis* were analyzed by difference-value of plasticity indexes (*DPI*) between field and hydroponics.

$$DPI={PI}_{field}-{PI}_{hydroponics}$$ (2)

*L*. *chinensis* functional trait data was transformed logarithmically (base 10). Model Type II regression was used to determine the slope (*a* = scaling exponent) and *y*-intercept (log_10_ *b*, where *b* is the allometric constant) of the log–log linear relationship. The software package *Standardized Major Axis Tests and Routines* [(S)MATR] was also used to determine whether the numerical value of *a* for the log–log plots differ between grazed and ungrazed plots [51]. (S)MATR was also used to provide the Model Type II equivalent of OLS standard analyses of covariance (ANCOVA). The significance level for testing slope heterogeneity was *P* < 0.05 (i.e. the notion of a common slope was rejected if *P* < 0.05). If the compared regressions have common slopes but different *y*-intercepts, then the difference in the *y*-intercepts might lead to a significant difference between the common slope obtained from [grazing exclusion] *vs* [grazing] or [clipping exclusion] *vs* [clipping].

All statistical analyses were performed to determine the significance of treatment means at *P* < 0.05 and *P* < 0.01 using SPSS 19.0 statistical software (SPSS, Inc., Chicago, IL). Allometric scaling was performed using the (S)MATR 2.1 software package. Statistical graphs were prepared using Sigmaplot ® 12.0 version (Systat Software, Inc., USA).

## Results

### *L*. *chinensis* functional traits responded to field clipping and grazing

**Fig 1:**
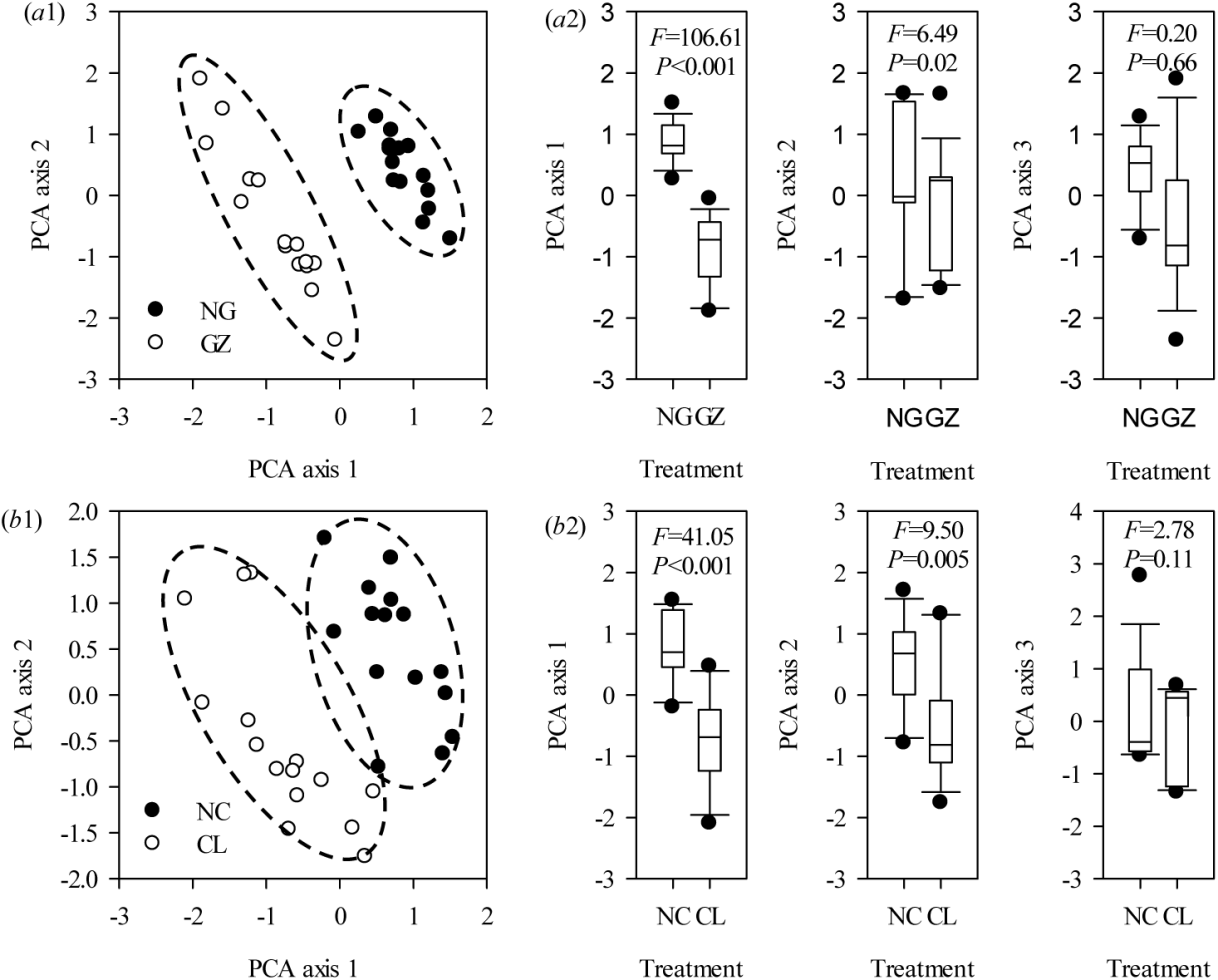
PCA bi-plot of *Leymus chinensis* (Trin.) Tzvelev functional traits for long-term grazing and clipping field treatments based on the variance in 10 functional traits explained by the first (PCA 1), second (PCA 2), and third (PCA 3) principal axes. (a1) and (b1): PCA ordination of *Leymus chinensis* (Trin.) Tzvelev plants from ungrazed or unclipped (solid circle) and grazed or clipped (empty circle) conditions in field and hydroponic experiments, respectively. (a2) and (b2): Box plots illustrate the score distribution of *Leymus chinensis* (Trin.) Tzvelev functional traits for plants from different communities along the three principal axes. Abbreviations: NC, unclipped; CL, clipped; NG, ungrazed; GZ, grazed.

**Fig 2:**
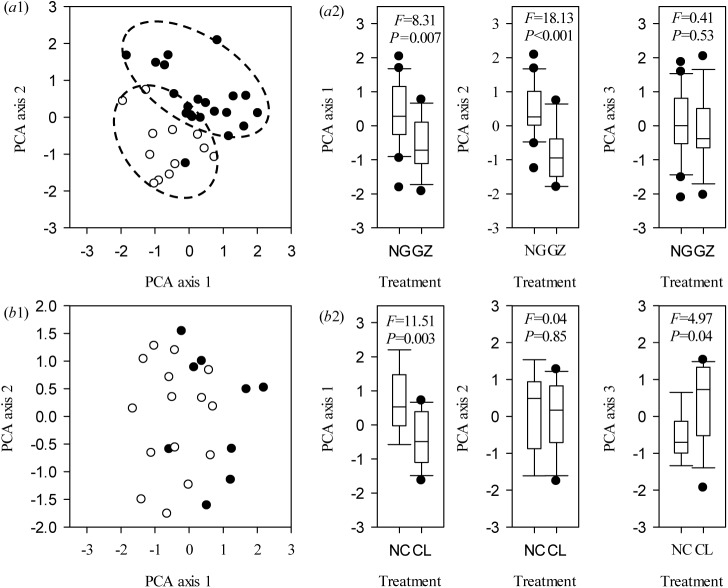
PCA bi-plot of *Leymus chinensis* (Trin.) Tzvelev functional traits for hydroponic treatment based on the variance in 10 functional traits explained by the first (PCA 1), second (PCA 2), and third (PCA 3) principal axes. (a1) and (b1): PCA ordination of *Leymus chinensis* (Trin.) Tzvelev plants from the ungrazed or unclipped (solid circle) and grazed or clipped (empty circle) treatments in hydroponic conditions, respectively. (a2) and (b2): Box plots illustrate the score distribution of *Leymus chinensis* (Trin.) Tzvelev functional traits from different communities along the three principal axes. Abbreviations are as described in Fig 1.

Long-term clipping and grazing had similar effects on the field functional traits of *L*. *chinensis*, with the majority traits significantly diminished by defoliation (Table 2; *P*<0.01). In addition, the majority traits were significantly correlated in clipping treatments (S1 Table) and grazing treatments (S2 Table). Traits associations between defoliated and non-defoliated treatments were analyzed (S1 Fig, S2 and S3 Tables). Defoliation due to grazing and clipping significantly decreased the loading score of plant size along PCA axis 1 and PCA axis 2 (Figs 1 and 2). Also, there were significant similar plasticity indexes for grazing and clipping experiments (*P*<0.01). In contrast, the values of plasticity indexes in grazing experiment were higher than clipping experiments (S2A Fig).

**Table 2:** Effects of long-term grazing and clipping on phenotypic traits of *Leymus chinensis* (Trin.) Tzvelev individuals in field experiments.

Plant traits	Grazing treatment (field)			Clipping treatment (field)		
	*F*-value	Effect	*P*-value	*F*-value	Effect	*P*-value
*LN*	1.32	**(0)**	0.26	22.18	**(-)**	<0.01
*LL*	1156.70	**(-)**	<0.01	374.85	**(-)**	<0.01
*LW*	129.39	**(-)**	<0.01	113.71	**(-)**	<0.01
*LLW*	340.28	**(-)**	<0.01	54.61	**(-)**	<0.01
*TLA*	181.15	**(-)**	<0.01	198.12	**(-)**	<0.01
*LA*	388.73	**(-)**	<0.01	240.09	**(-)**	<0.01
*SL*	893.08	**(-)**	<0.01	310.55	**(-)**	<0.01
*SD*	107.38	**(-)**	<0.01	6.24	**(-)**	0.02
*SLD*	658.41	**(-)**	<0.01	352.31	**(-)**	<0.01
*PH*	1306.80	**(-)**	<0.01	514.08	**(-)**	<0.01

Symbols

(-), treatments that had negative effects on phenotypic traits

(+), treatments that had positive effects on phenotypic traits

(0), treatments that had no effects on phenotypic traits.

Other abbreviations are the same as those in Table 1.

### Hydroponic testing on the morphological plasticity of *L*. *chinensis*

There were different characteristics of *L*. *chinensis* functional traits in response to clipping and grazing in the hydroponic experiment. The morphological plasticity of grazed *L*. *chinensis* which was maintained when clones were grown using hydroponics and was analyzed by ANOVA (Table 3) and PCA (Fig 2), respectively. However, plant functional traits such as *PH*, *LN*, *LL* and *SL* were not significantly different between the clipped and un-clipped treatments in hydroponic testing (Table 2, *P*>0.05). Clipping did not affect the loading score of plant size along PCA axis 2 (Fig 2), which was strongly associated with *PH*, *LN*, *LL* and *SL* (S8 Table).

**Table 3:** Effects of long-term grazing and clipping on phenotypic traits of *Leymus chinensis* (Trin.) Tzvelev individuals in hydroponic experiments. Abbreviations and symbols are the same as those in Table 1 and Table 2.

Plant traits	Grazing treatment (hydroponics)			Clipping treatment (hydroponics)		
	*F*-value	Effect	*P*-value	*F*-value	Effect	*P*-value
*LN*	8.59	**(-)**	0.01	1.58	**(0)**	0.22
*LL*	10.01	**(-)**	<0.01	1.00	**(0)**	0.33
*LW*	80.45	**(-)**	<0.01	43.38	**(-)**	<0.01
*LLW*	36.25	**(+)**	<0.01	6.72	**(+)**	0.02
*TLA*	46.85	**(-)**	<0.01	6.10	**(-)**	0.02
*LA*	43.11	**(-)**	<0.01	13.32	**(-)**	<0.01
*SL*	50.05	**(-)**	<0.01	0.32	**(0)**	0.58
*SD*	58.56	**(-)**	<0.01	11.65	**(-)**	<0.01
*SLD*	2.56	**(0)**	0.12	8.43	**(+)**	0.01
*PH*	58.62	**(-)**	<0.01	0.42	**(0)**	0.52

### Partial memory effect on the morphological plasticity of *L*. *chinensis*

**Fig 3:**
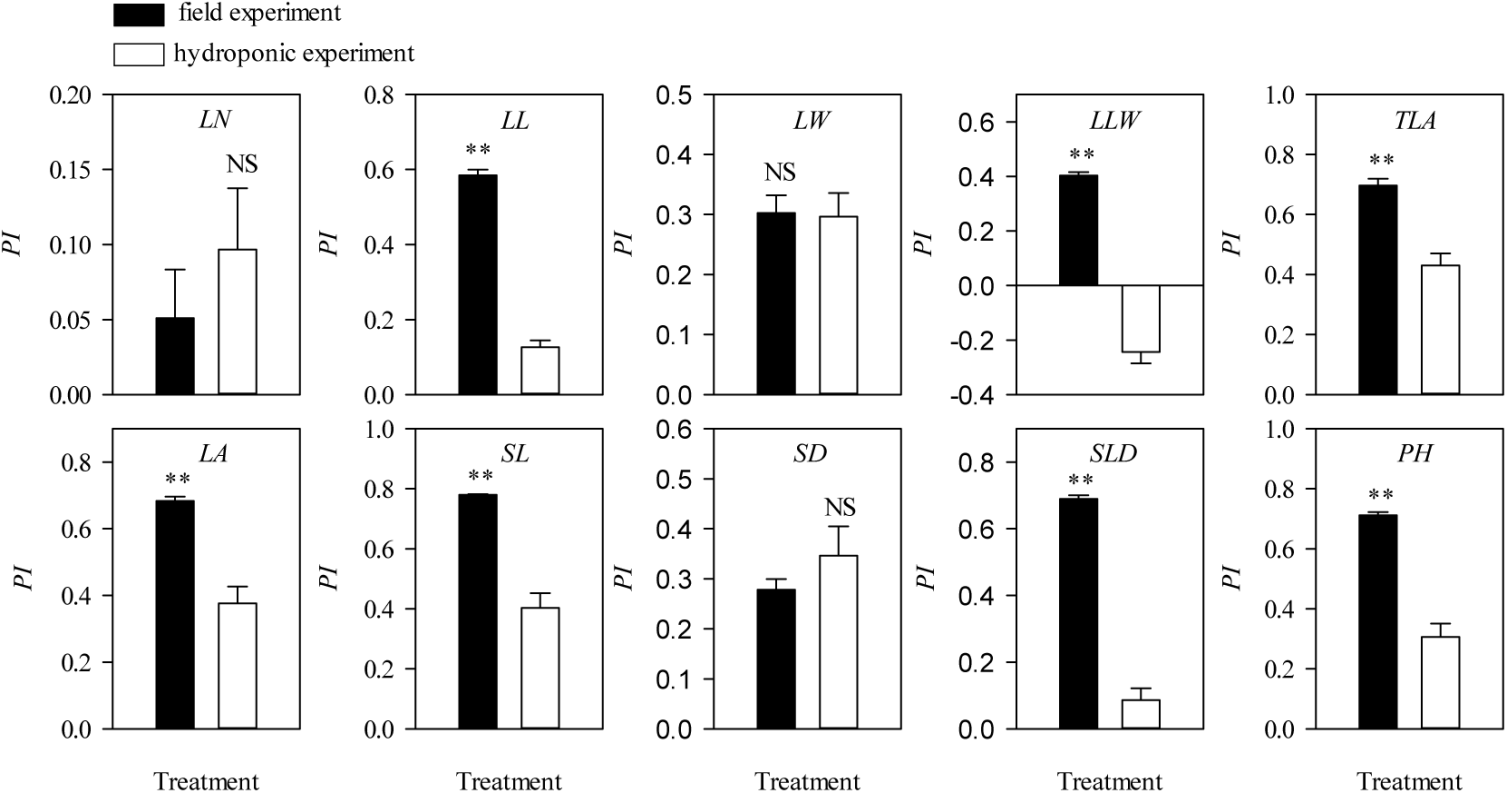
Differences in the plasticity index of *Leymus chinensis* (Trin.) Tzvelev phenotypic traits between field grazing and hydroponic conditions. Symbols: **, *P* < 0.01; *, *P* < 0.05; NS, *P* > 0.05.

**Fig 4:**
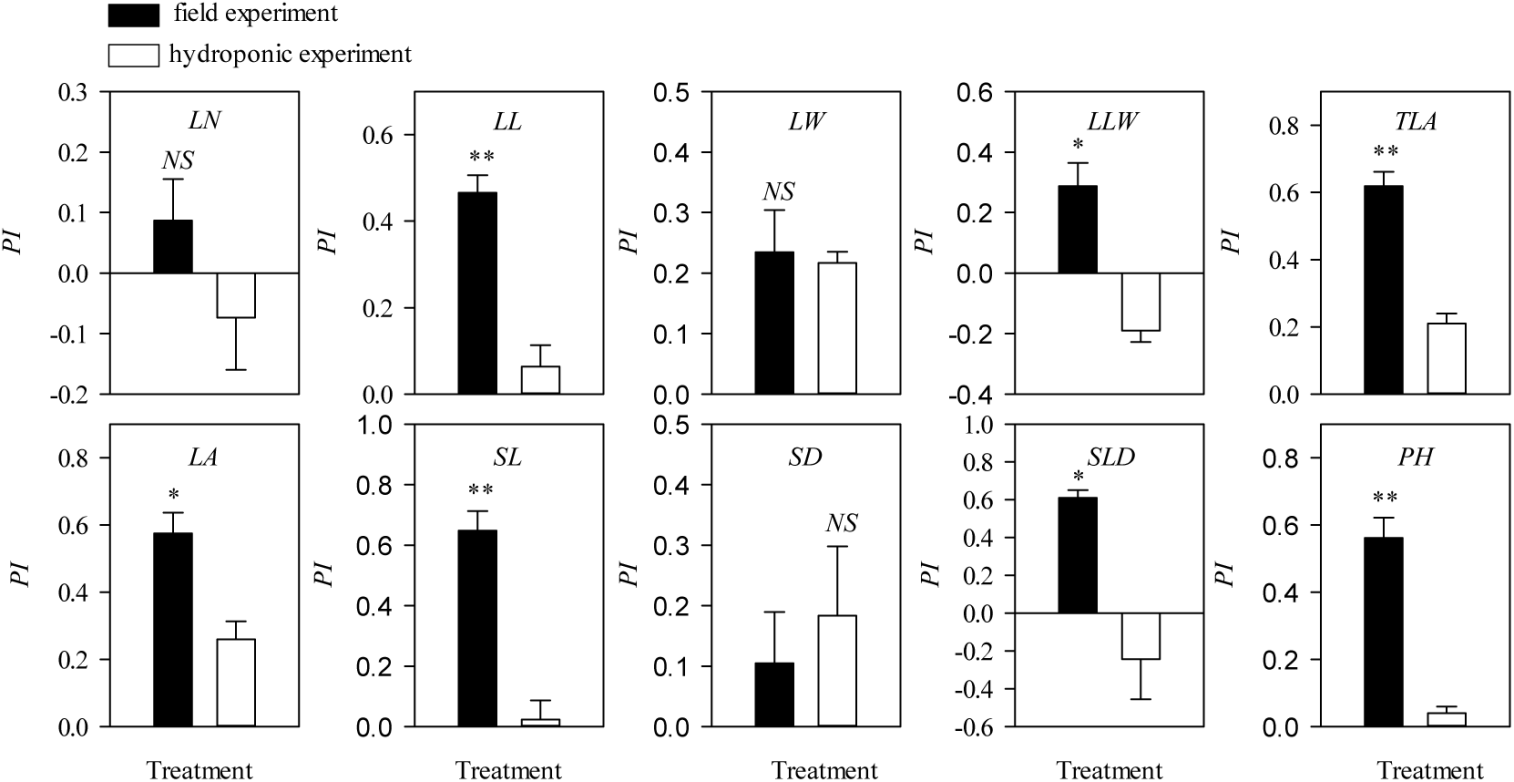
Differences in the plasticity index of *Leymus chinensis* (Trin.) Tzvelev phenotypic traits between field clipping and hydroponic conditions. Symbols are as described in Fig 3.

**Fig 5:**
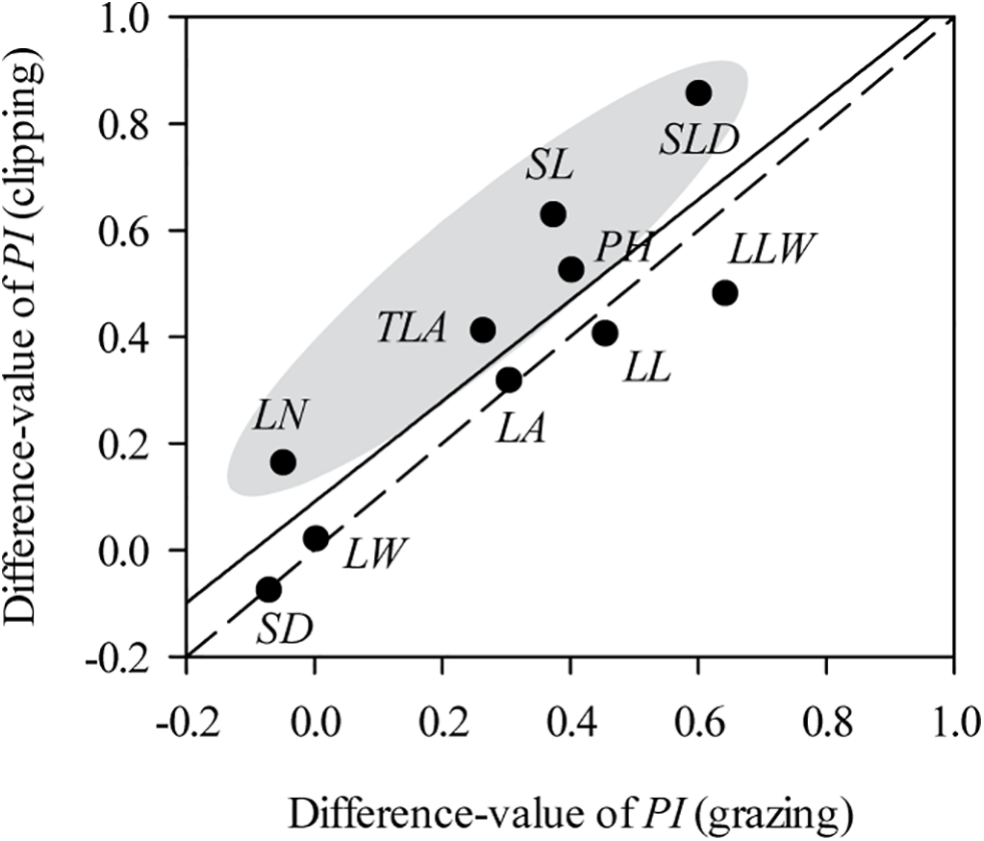
Relationships between the difference-value of plasticity index (*PI*) of grazing and clipping treatments. Difference-value of *PI*: *PI* (*in situ*)–*PI* (hydroponics). The correlation between the differences in *PI* was tested by Pearson’s method (*r* = 0.87, *P* < 0.01). The gray area represents traits that exhibit a larger change in *PI* after clipping treatments than grazing treatments. Solid line: linear fit; dashed line: 1:1 line.

Values for plasticity indexes values of *L*. *chinensis* traits grown under hydroponic conditions were dramatically lower than for field data in both the grazing (S2C Fig) and clipping (S2D Fig) experiments. Specifically, for grazed treatments, some key *L*. *chinensis* traits, such as *LL*, *LA*, *SL* and *PH*, were significantly higher in field testing (*P*<0.05, Fig 3) with a similar tendency in clipped treatments (Fig 4). Moreover, the difference-value of plasticity indexes was higher in grazed treatments than in clipping treatments (Fig 5).

### Allometric scaling of stem-leaf with individual size variations

**Fig 6:**
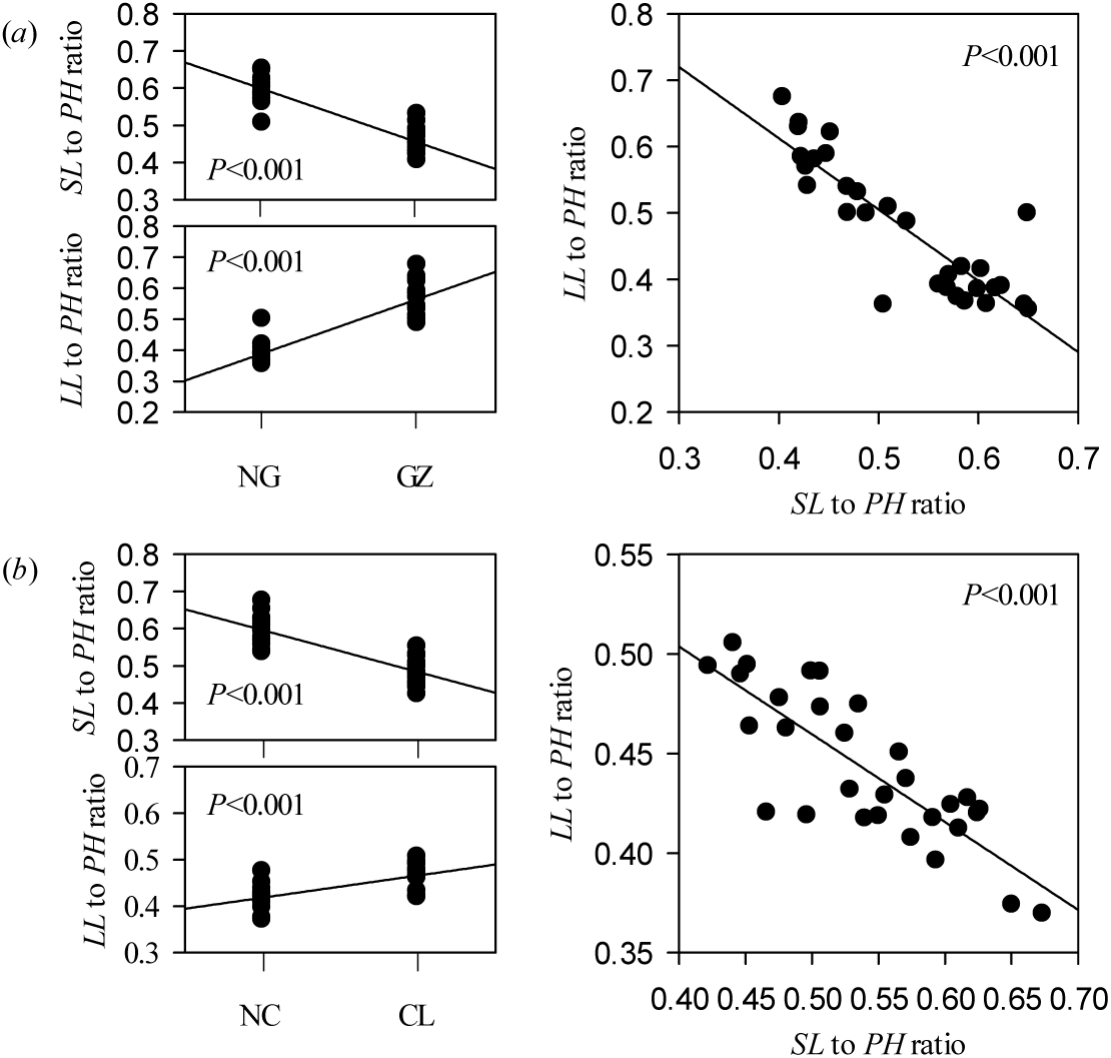
Trade-offs between the stem length (*SL*) to plant height (*PH*) ratio and the leaf length (*LL*) to *PH* ratio affected by long-term grazing (*a*) and clipping (*b*) treatments in field habitats. The correlations between the *SL* to *PH* ratio or the *LL* to *PH* ratio and grazing or clipping were tested using Spearman’s method. Trade-offs between the *SL* to *PH* ratio and the *LL* to *PH* ratio were tested using Pearson’s method. Abbreviations are as described in Fig 1 and Table 1.

**Fig 7:**
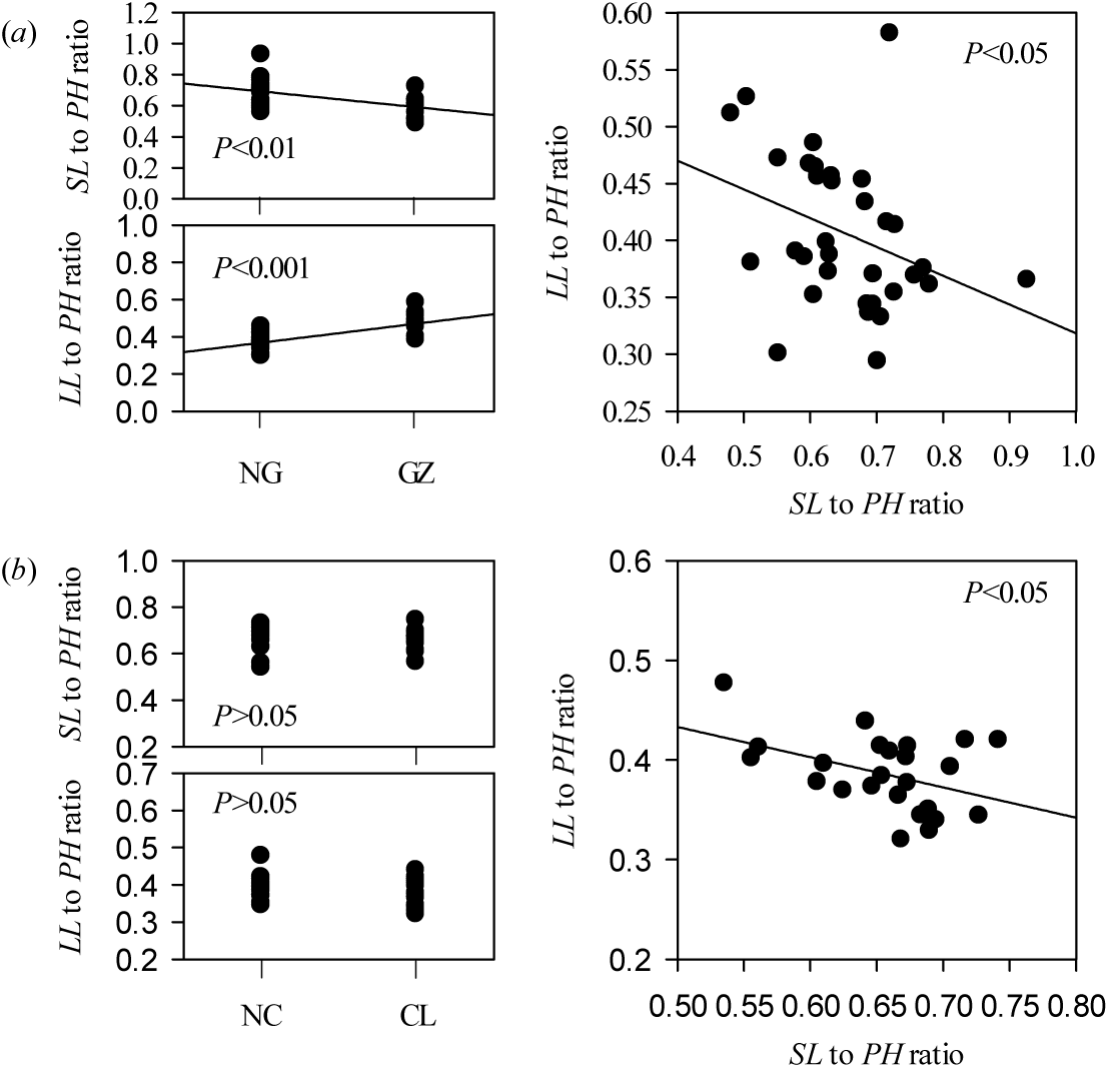
Trade-offs between the *SL* to *PH* ratio and the *LL* to *PH* ratio affected by long-term grazing (*a*) and clipping (*b*) treatments in hydroponic habitats. The correlations between the *SL* to *PH* ratio or the *LL* to *PH* ratio and grazing or clipping treatment were tested by Spearman’s method. The trade-offs between the *SL* to *PH* ratio and the *LL* to *PH* ratio were tested using Pearson’s method. Abbreviations are as described in Fig 1 and Table 1.

In the field and hydroponic experiments, response of *L*. *chinensis* morphological traits to clipping and grazing could be categorized as either sensitive (mostly in stem traits) or insensitive (mostly in leaf traits) traits (S2 Fig, Fig 3, Fig 4). As a result of defoliation, *SL* to *PH* ratio was decreased significantly, whereas *LL* to *PH* ratio was increased significantly in field testing (*P*<0.01). There was a highly significantly negative trade-off between the *SL* to *PH* ratio and the *LL* to *PH* ratio (Fig 6). However, in the hydroponic experiment these responses were maintained only in the grazing comparison and not the clipping comparison (Fig 7). More specifically, the relationships for leaf and stem (log-log scaling) showed that significant allometric relationships existed in the four treatment combinations (*P*<0.01). *SMA* tested for common slopes reveal no significant difference in the slopes for the relationships of *PH* vs. *LL*, *PH* vs. *LW* and *LL* vs. *SL* exhibited by the four treatment combinations (*P*>0.05). However, the allometric scaling was significantly shifted by grazing or clipping disturbances (Tables 4, 5 and 6).

**Table 4:** Standardized major axis (*SMA*) regression slopes and confidence intervals (*CI*) for log-log transformed relationships between plant height and leaf length of *Leymus chinensis* (Trin.) Tzvelev in grazing exclusion and grazing groups. 95% *CI* of *SMA* slopes, intercepts, and common slopes are shown. In several bivariate cases, *SMA* tests for common slopes revealed no significant differences between the two groups (i.e., *P* > 0.05). In such cases, common slopes and *CI*s for the bivariate relationships are shown. Significant shifts along a common slope are indicated.

Experiment	Group	R^2^	Slope	Intercept	Common slope	Shift?
Field	NG	0.00^NS^	1.19 (0.67, 2.10) ^NS^	0.13 (-0.95, 1.20) ^NS^	1.47 (1.04, 2.05) ^NS^	Yes
	GZ	0.48^**^	1.64 (1.08, 2.49)^*^	-0.47 (-1.27, 0.33) ^NS^		
	NC	0.36^*^	0.97 (0.61, 1.55) ^NS^	0.42 (-0.25, 1.08) ^NS^	1.04 (0.77, 1.39) ^NS^	Yes
	CL	0.53^**^	1.09 (0.73, 1.62) ^NS^	0.23 (-0.29, 0.75) ^NS^		
Lab	NG	0.22^*^	0.86 (0.57, 1.29) ^NS^	0.66 (0.10, 1.22)^*^	1.19^*^	
	GZ	0.72^**^	1.49 (1.04, 2.15)^*^	-0.40 (-1.23, 0.43) ^NS^		
	NC	0.28^NS^	0.89 (0.44 1.79) ^NS^	0.58 (-0.47, 1.64) ^NS^	0.85 (0.67, 1.09) ^NS^	No
	CL	0.80^**^	0.84 (0.65, 1.10) ^NS^	0.66 (0.31, 1.01)^**^		

Symbols

**, *P* < 0.01

*, *P* < 0.05

NS, *P* > 0.05.

Abbreviations are the same as those in Fig 1 and Table 1.

**Table 5:** Standardized major axis (*SMA*) regression slopes and their confidence intervals for the log-log transformed relationship between *PH* and *SL* of *Leymus chinensis* (Trin.) Tzvelev in grazing exclusion and grazing groups. Symbols and abbreviations are as described in Table 1, Fig 1 and Fig 4.

Experiment	Group	R2	Slope	Intercept	Common slope	Shift?
Field	NG	0.47^*^	0.74 (0.48, 1.13) ^*^	0.67 (0.12, 1.22) ^*^	0.70 (0.60, 0.83) ^NS^	Yes
	GZ	0.91^**^	0.70 (0.58, 0.84) ^**^	0.65 (0.52, 0.79) ^**^		
	NC	0.75^**^	0.60 (0.80) ^**^	0.86 (0.58, 1.14) ^**^	0.64 (0.52, 0.79) ^NS^	Yes
	CL	0.72^**^	0.68 (0.50, 0.93) ^*^	0.69 (0.44, 0.95) ^**^		
Lab	NG	0.59^**^	0.56 (0.41, 0.75) ^**^	0.97 (0.66, 1.28) ^**^	0.68 (0.52, 0.84) ^NS^	Yes
	GZ	0.79^**^	0.81 (0.59, 1.11) ^NS^	0.53 (0.12, 0.94) ^*^		
	NC	0.78^**^	0.52 (0.35, 0.79) ^*^	1.04 (0.65, 1.44) ^*^	0.95^*^	
	CL	0.87^**^	1.06 (0.85, 1.32) ^NS^	0.08 (-0.34, 0.49) ^NS^		

**Table 6:** Standardized major axis (*SMA*) regression slopes and their confidence intervals for the log-log transformed relationship between stem length and leaf length of *Leymus chinensis* (Trin.) Tzvelev in grazing exclusion and grazing groups. Symbols and abbreviations are as described in Table 1, Fig 1 and Fig 4.

Experiment	Group	R2	Slope	Intercept	Common slope	Shift?
Field	NG	0.04^NS^	1.61 (0.92, 2.81)^**^	-0.73 (-2.16, 0.69)^NS^	1.99 (1.36, 2.88)^NS^	Yes
	GZ	0.25 ^NS^	2.34 (1.42, 3.86)^**^	-1.60 (-2.97, -0.23)^*^		
	NC	0.07 ^NS^	1.63 (0.94, 2.82)^**^	-0.73 (-2.07, 0.60)^NS^	1.61 (1.11, 2.33)^NS^	Yes
	CL	0.20 ^NS^	1.60 (0.95, 2.67)^**^	-0.68 (-1.69, 0.32)^NS^		
Lab	NG	0.25^*^	1.54 (1.03, 2.30)^**^	-0.56 (-1.54, 0.43)^NS^	1.72 (1.31, 2.21)^NS^	Yes
	GZ	0.75^**^	1.84 (1.30, 2.60)^**^	-1.14 (-2.10, -0.19)^*^		
	NC	0.08 ^NS^	1.70 (0.78, 3.70)^**^	-0.88 (-3.18, 1.42)^NS^	0.91 (0.65, 1.30)^NS^	No
	CL	0.62^**^	0.80 (0.55, 1.14)^**^	0.55 (0.10, 1.01)^*^		

## Discussion

### Morphological plasticity of *L*. *chinensis*

*L*. *chinensis* is a dominated species in Inner Mongolia grassland and tends to decrease with increasing grazing pressure [21, 52]. Our results showed that the majority of phenotypic traits of *L*. *chinensis* tended to miniaturize in response to long-term disturbance from clipping and grazing under field conditions. Previous studies reported that the size of many plants was reduced under continuous grazing pressure [36, 53], supporting our results. However, these previous studies did not investigate the effects of defoliation by long-term clipping on plant growth and phenotypic responses [54]. Our results indicated that there was some similarity in the change of functional traits in response to both clipping and grazing under the field conditions.

Plant functional traits which link to ecosystem function were the basic elements adapted to clipping and grazing [55]. In our results, defoliation reduced leaf number, leaf length, leaf width, leaf area, stem length, stem diameter, and plant height in this semiarid grassland ecosystem. Other studies have found that not all the grassland plant species had the same morphological plasticity with clear distinctions between grazing-susceptible and grazing-resistant species in response to long-term defoliation [17, 56]. Since *L*. *chinensis* appears to be more susceptible in grassland population in typical steppe in eastern Eurasian temperate grassland [53, 57], miniaturization of *L*. *chinensis* would significantly affect the structure and function of the grassland ecosystem. The change in plant functional traits can cause a cascade reactions from individual, species, population to ecosystem [58, 59].

The ability of grassland to produce biomass is central to both ecosystem function and their usefulness to supply forage for grazing animals [60]. Many studies have identified loss of biodiversity as a key mechanism to explain productivity decline [61]. How plant traits at the species level change in response to long-term heavy defoliation, which is an important process that influences grassland productivity [5]. Our results demonstrated that both grazing and clipping had significant effects on *L*. *chinensis* leaf and stem functional traits. Size of individual *L*. *chinensis* plants was significantly reduced compared with un-defoliated plants in the native grassland community. Further, other phenotypic traits associated with the aboveground biomass of individual plants also declined with grazing and clipping. Since the morphological plasticity of *L*. *chinensis* was impacted by the disturbance of grazing we believe this is a key process which contributes to declines in grassland productivity.

### Different effects of clipping and grazing

In Mongolia grasslands, grazing is the main form of defoliation, with some clipping occurring in hay production areas. Both defoliation processes can alter the function of ecosystems and several previous studies have used clipping to simulate grazing effects [25]. There are three main processes associated with grazing impacts on ecosystems: biomass removal; trampling; and deposition of excrement [62]. To some extent, the effect of clipping is similar to biomass removal by grazing animals, but is less selective and more spatially uniform [28]. Therefore grazing is not completely replicated by clipping because the influence of trampling and excrement are not taken into accounted.

Nevertheless, we observed some similarities between clipping and grazing under field conditions. Firstly, the direction of morphological plasticity was similar with both long-term clipping and grazing, diminishing leaf traits, stem traits and the whole plant traits compared with plants from un-defoliated populations. Secondly, the sensitivity of traits was similar with sensitive (e.g. plant height) and unresponsive (e.g. leaf number, leaf width, stem diameter) traits responding similarly to clipping and grazing. There was also a highly significant correlation for all functional plasticity changes between grazing and clipping. Thirdly, though the effects of the two disturbances were different, the tradeoffs for plant allocation to stems or leaves were similar. Thus, these results implied that the traits and tradeoffs were similar under the different forms of defoliation and plants responded to the stress of defoliation in a similar way [63].

However, the response of *L*. *chinensis* plants grown under hydroponic conditions when compared to the field experiment was weaker for grazing comparisons while no responses was observed for clipping. The results demonstrated that the effects of clipping were mainly related to nutrition and water, rather than the adaption of plants traits which is more likely to have occurred with grazing. Even when plant growth is not limited by resources (nutrition, water etc.), other factors such as saliva, intake, selective grazing, trampling and, excrement which are all part of the grazing process will also reduce plant growth rate [64]. Since *L*. *chinensis* is a preferred species for large herbivores [65] it undoubtedly sustains higher relative levels of defoliation. Moreover, in this study difference in duration between the grazing treatments (continuous three decades) and clipping treatments (continuous two decades) may be a potential reason in the contrasting effects of long-term grazing and clipping on plant morphological plasticity in typical steppe. However, to date there is not sufficient experimental evidence in molecular ecology to understand the contrasting processes of *L*. *chinensis* miniaturization due to dwarf resulting from grazing and clipping.

### Allometry in different functional traits

Our results demonstrated that all of the *L*. *chinensis* functional traits had an allometry that responds to defoliation by clipping and grazing. Some traits such as stem biomass, aboveground biomass and plant height were sensitive traits. Yet other traits such as leaf number, leaf mass per area, leaf biomass, leaf width, stem diameter did not respond to defoliation. Hence, the functional traits were different in their response mechanism to biotic and abiotic disturbances [66].

Leaf traits which have many important functions are recognized as the key traits in plants. Zheng et al. (2010) reported that while leaves became smaller, leaf number increased in response to long-term grazing [11]. Our results contradict this finding as leaf number decreased with defoliation. The reason our results didn’t support Zheng’ may be two reasons. Firstly, the plant species were different in the two studies. The leaf traits of different species can have different characteristics that respond differently to defoliation. Secondly, the utilization history of the two experimental sites may have affected the relationship with long-term defoliation may have altered the ability of plants to increase leaf number. Thirdly, sampling methods of transect and plots were used differed in the two studies. The allometry between leaf number and leaf size relied on species, grazing history and ecological scale.

In addition, our study also indicated that leaf length/width, stem length/diameter and leaf mass per area decreased with defoliation. Some relevant previous studies supported our opinion that these traits relate to plant photosynthesis [12]. Laliberte et al. (2012) found that leaves became thinner and plants had low leaf mass per area (LMA) in the grazed environment compared with the ungrazed native grassland [67]. The allometry in plant leaf, stem phenotypic traits and biomass traits in long-term clipping and grazing implies that the ecological strategies changes photosynthesis to adapt to new environments [11].

### Potential mechanisms in the adaptation to grazing

The results from the laboratory experiment indicate it is possible for the dwarf memory of *L*. *chinensis* individuals produced by asexual reproduction to adapt to long-term grazing. More importantly, our results indicated a partial and slight memory effect that was not a result of a different environment in the controlled environment experiment compared with the results of grazing and clipping treatments, respectively. Since the limitation of resources such as nutrition, water and light was not the only or main factors driving the miniaturization process by grazing, we are presume that some epigenetic and physiological mechanisms are contributing to the process of morphological plasticity. Research on the restoration process of grasslands degraded by overgrazing indicates that plant individuals could return to normal after several years of grazing exclusion [68]. This implies that the dwarf memory of *L*. *chinensis* due to grazing may not a heritable variation and raises further questions as to what the mechanism controls dwarf memory in a grazed habitat and how it may be in the restoration process, and why the same mechanism does not appear to be the same between grazing and clipping.

The phenotypic characteristic maintained in vegetative propagation might be the grazing avoidance mechanism that forms part of adaptation to grazing [69]. Although in this study we observed that the dwarf memory of *L*. *chinensis* phenotype due to grazing was maintained by asexual reproduction, there are only a small number of studies that relate dwarf memory of grazing effects in grassland plants that reproduce by seeds. As early as 1990s, McKinney & Fowler (1991) identified genetic adaptations to grazing in the grass *Cenchrus incertus* [31] when seeds from long-term grazed and ungrazed populations were germinated and grown in a common greenhouse, maintain differences in the second generation. Hence, the morphological differentiation among plants with different grazing histories appears to be the outcome of a phenotypically plastic response of adapted genotypes [70]. Moreover, many previous studies showed that the ungrazed individuals were not significantly genetically differentiated from the grazed individuals based on molecular marker analysis. [71, 72]. Therefore, the grazing avoidance mechanism is codetermined by environmental conditions and genetics with the genetic variation most likely to be at an epigenetic level [73].

## Conclusions

We found that the majority of *L*. *chinensis* phenotypic traits tended to miniaturize in response to long-term defoliation by clipping and grazing under field conditions. The phenotypic traits, of the leaf and stem, and the whole aboveground plant biomass all decreased significantly. There was significant similarity of all functional plasticity and tradeoffs between grazing and clipping in the field experiment. However, these traits were not expressed in the hydroponic experiment. The significant difference was maintained in comparisons between grazed and ungrazed populations. Yet there was no significant difference between clipped and unclipped populations. These results demonstrated that the morphological plasticity of *L*. *chinensis* induced by artificial clipping was different with that by livestock grazing. The miniaturization of plant size in long-term grazed grassland may reflect retained characteristics of dwarf memory for adaptation to long-term grazing by large herbivores.

## Supporting Information

S1 Fig The contribution of five axes determined by a principal component analysis to the variance in *Leymus chinensis* (Trin.) Tzvelev traits. (a) Grazing and non-grazing treatments in field experiments; (b) clipping and unclipping treatments in field experiments; (c) grazing and non-grazing treatments in hydroponic experiments; (d) clipping and non-clipping treatments in hydroponic experiments.
(TIF)

S2 Fig Relationships among the plasticity indexes (*PI*s) of traits for grazing and clipping treatments in field and hydroponic habitats. Solid line: linear fit; dashed line: 1:1 line.
(TIF)

S3 Fig Rank order of plasticity indexes for *Leymus chinensis* (Trin.) Tzvelev functional traits. (a) grazing–field; (b) grazing–hydroponics; (c) clipping–field; (d) clipping–hydroponics.
(TIF)

S1 Table Relationships among contrasting functional traits of *Leymus chinensis* (Trin.) Tzvelev in a field clipping experiment. Abbreviations: *PH*, Plant height; *LN*, Leaf number; *LL*, Leaf length; *LW*, Leaf width; *LLW*, Leaf length to width ratio; *LA*, Leaf area; *SL*, Stem length; *SD*, Stem diameter; *SLD*, Stem length to diameter ratio. Symbols: **, *P* < 0.01; *, *P* < 0.05, NS, *P* > 0.05.
(XLSX)

S2 Table Relationships among contrasting functional traits of *Leymus chinensis* (Trin.) Tzvelev in a field grazing experiment. Abbreviations and symbols are as described in S1 Table.
(XLSX)

S3 Table Relationships among contrasting functional traits of *Leymus chinensis* (Trin.) Tzvelev for field clipped and unclipped conditions in a hydroponics experiment. Abbreviations and symbols are as described in S1 Table.
(XLSX)

S4 Table Relationships among contrasting functional traits of *Leymus chinensis* (Trin.) Tzvelev from field grazed and ungrazed conditions in a hydroponics experiment. Abbreviations and symbols are as described in S1 Table.
(XLSX)

S5 Table Correlations among principal component scores for five axes and contrasting plant traits of *Leymus chinensis* (Trin.) Tzvelev individuals in a field grazing experiment. Abbreviations and symbols are as described in S1 Table.
(XLSX)

S6 Table Correlations among principal component scores for five axes and contrasting plant traits of *Leymus chinensis* (Trin.) Tzvelev individuals in a field clipping experiment. Abbreviations and symbols are as described in S1 Table.
(XLSX)

S7 Table Correlations among principal component scores for five axes and ten plant traits of *Leymus chinensis* (Trin.) Tzvelev for field grazing and non-grazing conditions in a hydroponics experiment. Abbreviations and symbols are as described in S1 Table.
(XLSX)

S8 Table Correlations among principal component scores for five axes and the contrasting plant traits of *Leymus chinensis* (Trin.) Tzvelev individuals for field clipping and non-clipping conditions in a hydroponics experiment. Abbreviations and symbols are as described in S1 Table.
(XLSX)
