# Supplementary figures and images for: Contrasting Effects of Long-Term Grazing and Clipping on Plant Morphological Plasticity: Evidence from a Rhizomatous Grass
Source: PLoS One. 2015 Oct 27;10(10):e0141055. doi: 10.1371/journal.pone.0141055 (PMC4624235; doi:10.1371/journal.pone.0141055)

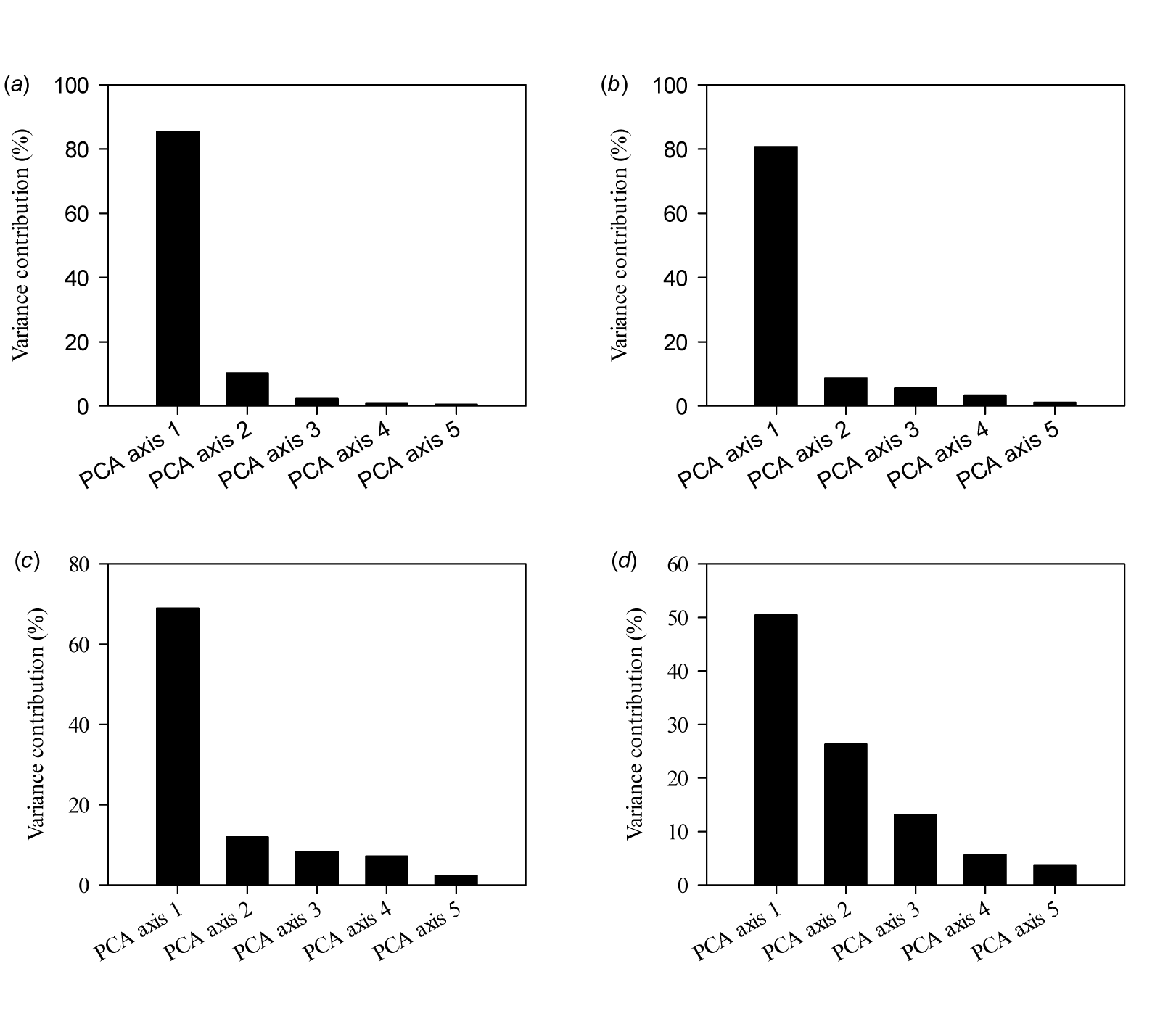

Supplement: S1 Fig — (a) Grazing and non-grazing treatments in field experiments; (b) clipping and unclipping treatments in field experiments; (c) grazing and non-grazing treatments in hydroponic experiments; (d) clipping and non-clipping treatments in hydroponic experiments. (TIF) [file pone.0141055.s001.tif]

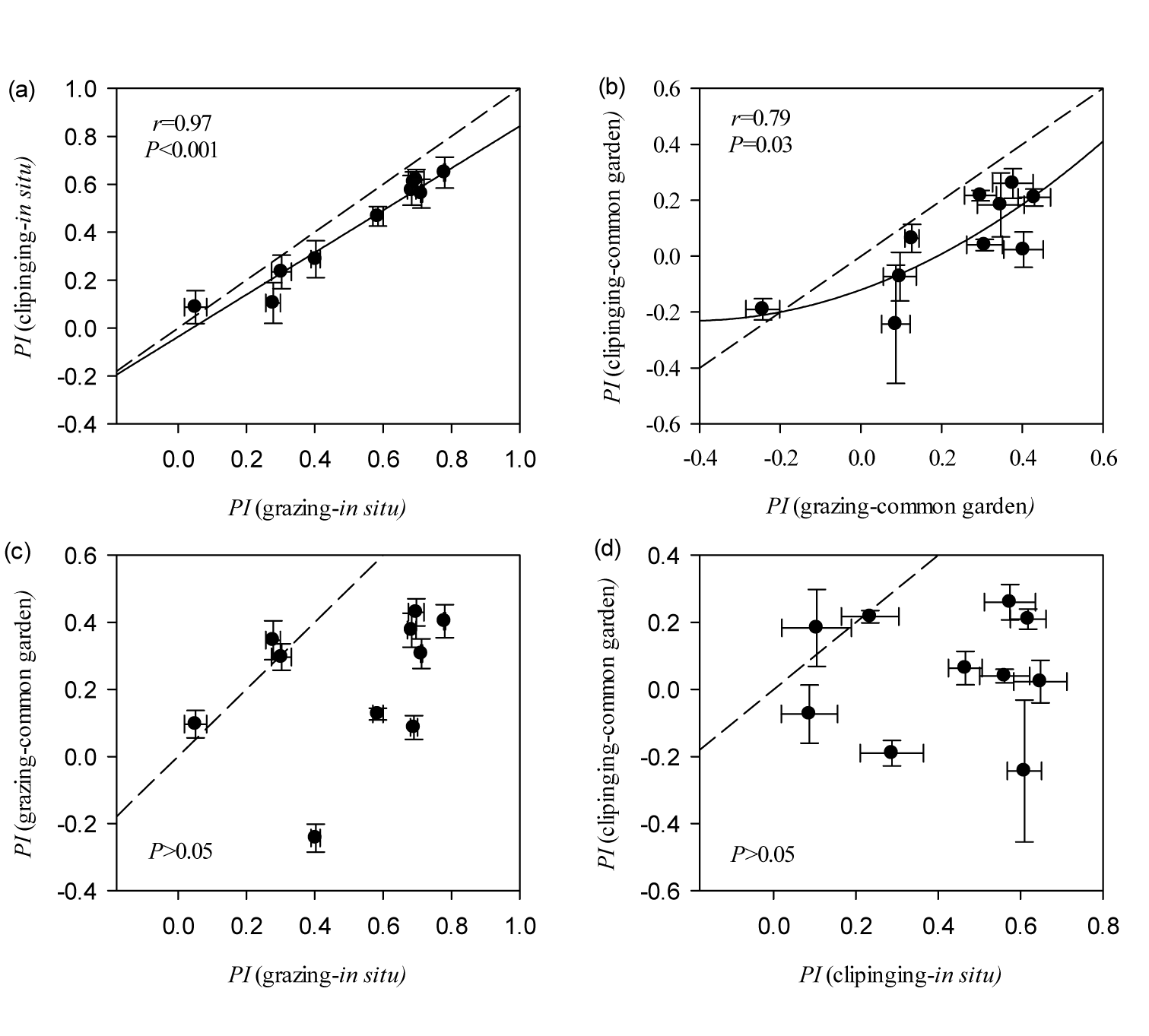

Supplement: S2 Fig — Solid line: linear fit; dashed line: 1:1 line. (TIF) [file pone.0141055.s002.tif]

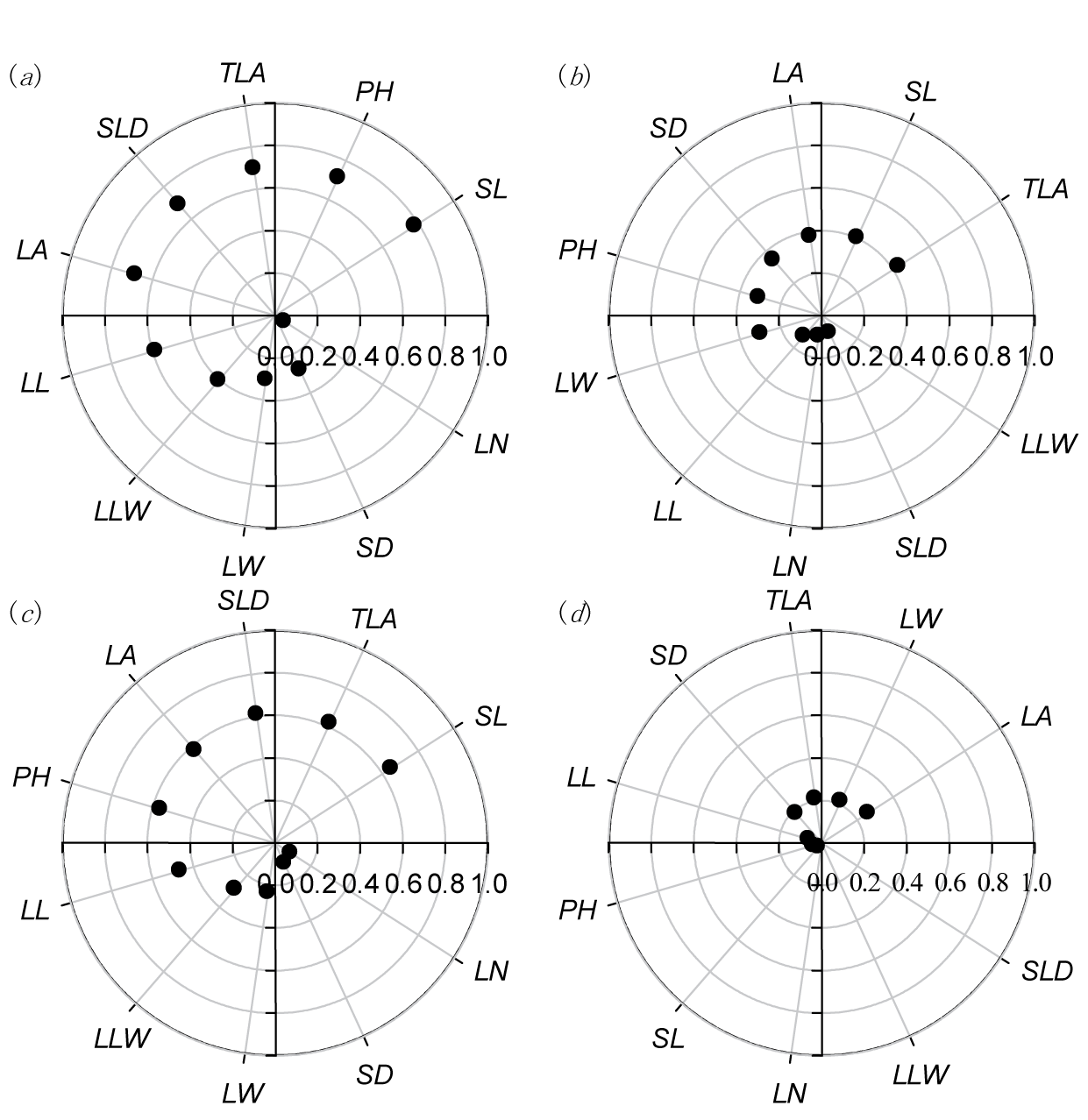

Supplement: S3 Fig — (a) grazing–field; (b) grazing–hydroponics; (c) clipping–field; (d) clipping–hydroponics. (TIF) [file pone.0141055.s003.tif]
